# High Performance Graphene Nano-ribbon Thermoelectric Devices by Incorporation and Dimensional Tuning of Nanopores

**DOI:** 10.1038/srep11297

**Published:** 2015-06-17

**Authors:** Md Sharafat Hossain, Feras Al-Dirini, Faruque M. Hossain, Efstratios Skafidas

**Affiliations:** 1 Department of Electrical and Electronic Engineering, University of Melbourne, VIC 3010, Australia; 2 Victorian Research Laboratory, National ICT Australia, VIC 3010, Australia; 3 Centre for Neural Engineering, University of Melbourne, VIC 3010, Australia

## Abstract

Thermoelectric properties of Graphene nano-ribbons (GNRs) with nanopores (NPs) are explored for a range of pore dimensions in order to achieve a high performance two-dimensional nano-scale thermoelectric device. We reduce thermal conductivity of GNRs by introducing pores in them in order to enhance their thermoelectric performance. The electrical properties (Seebeck coefficient and conductivity) of the device usually degrade with pore inclusion; however, we tune the pore to its optimal dimension in order to minimize this degradation, enhancing the overall thermoelectric performance (high ZT value) of our device. We observe that the side channel width plays an important role to achieve optimal performance while the effect of pore length is less pronounced. This result is consistent with the fact that electronic conduction in GNRs is dominated along its edges. Ballistic transport regime is assumed and a semi-empirical method using Huckel basis set is used to obtain the electrical properties, while the phononic system is characterized by Tersoff empirical potential model. The proposed device structure has potential applications as a nanoscale local cooler and as a thermoelectric power generator.

Graphene, a two-dimensional (2D) allotrope of carbon, has attracted much attention due to its superior electrical, mechanical and thermal properties^1^,^2^,^3^. Additionally, techniques like the application of a gate potential, introduction of controlled defects and disorders, and different types of electrical confinement open the possibility of modulating and further enhancing its physical properties^4^,^5^. One potentially promising application for graphene could be in the field of thermoelectronics. The performance of a thermoelectric device described by its thermoelectric figure of merit (ZT), depends on electrical conductance (*σ*), Seebeck coefficient (S) and thermal conductance(*k*) according to the relation, *ZT* = *S*^2^*σT/k*. Limited attention has been paid to graphene as a potential thermoelectric material primarily due to its high thermal conductivity, however, in graphene there are some permissible tradeoffs among these parameters that can be used to greatly reduce this high thermal conductivity while maintaining its very high electrical conductivity, leading to greatly enhanced performance.

In the mid-1990s, it was theoretically predicted that improvement in thermoelectric performance can be achieved in nanostructured materials due to phonon boundary scattering and lateral quantum confinement of electrons^6^. This work resulted in several subsequent experimental reports that proved the concept. For example, by nanostructuring silicon into nanowires researcher could achieve a 100 fold improvement in ZT^7^,^8^. Similarly, bulk nanostructured materials and nanoscale complex materials exhibited enhanced thermoelectric performance^9^,^10^. Graphene nano-ribbon (GNR), a quasi-one-dimensional system, which has proven to have many interesting properties such as band-gap opening^11^, is a potential candidate for nanoscale thermoelectronics. Huskin *et al*. showed theoretically that thermal and electrical properties of graphene can be controlled independently through defect engineering^12^. Several theoretical work has been carried out to investigate the performance of graphene and graphene nanoribbons as thermoelectric materials. Ouyang *et al*.^13^ have studied the thermoelectric properties of pristine GNRs. Although high thermopower can be achieved when using armchair GNRs, pristine GNRs are considered an inefficient thermoelectric material with achievable figures of merit ranging from 0.05 to 0.2, which are considered relatively low. This is primarily due to the high thermal conductance of pristine GNRs. Researchers have theoretically investigated many different methods to reduce the thermal conductivity of GNRs without reducing their electrical performance, including: Antidot lattices^14^, edge disorders^15^, edge passivation^16^, random hydrogen vacancies^17^, doping carbon isotopes^18^, mechanical strain^19^, superlattices^20^, GNR junctions, and nanopore GNRs^12^,^21^. Cheng *et al*. proposed the use of heavy adatoms and nanopores to enhance the thermoelectric property of GNRs, predicting a ZT value of 3 at low temperature (T = 40 K)^22^. Armchair edged GNRs with antidots were predicted to achieve a ZT of 0.25^14^. Mazzamuto *et al*. have proposed the use of the resonant tunneling effect of mixed edged GNRs and predicted a ZT value above unity^23^. More recently, Liang *et al*.^24^ proposed a new structure, nanowiggles, which also exploited the resonant tunneling effect to enhance thermoelectric performance. Their devices were predicted to achieve a ZT of 0.79 at room temperature. Yang *et al*.^20^ proposed a hybrid graphene/boron nitride structure to enhance the thermoelectric property and envisaged a 1.5–3 times enhancement for semiconducting GNRs. Mazzamuto *et al*.^25^ have also theoretically investigated edge disorders in armchair GNRs, but although thermal conductance can be reduced, edge roughness affected the electrical performance as well, having an overall negative impact on ZT. On the other hand, Sevincli *et al*.^26^ have predicted that by introducing edge roughness a ZT of 4 can be achieved for zigzag GNRs. Similarly, Chang *et al*.^21^ have proposed the use of GNRs with periodic arrays of nanopores for long structures. Their device, of length 1.2 *μ*m, was predicted to achieve a ZT of 2 at room temperature. The reduced thermal conductivity of their device can be attributed to the use of long wires of the order of micrometers. Although the above mentioned work is theoretical with no experimental validation other groups have experimentally investigated the thermopower of graphene^27^,^28^,^29^.

Here we propose a new structure based on a GNR nanopore thermoelectric device with dimensions of a few nanometers and with significantly enhanced thermoelectric performance. It has previously been predicted in theory that local charge current profile peaks around the GNR edges^21^,^30^. By varying the number of atoms in the side edges (side channels) we could achieve superior electrical performance, while the introduction of the pore greatly reduce the thermal conductivity. These two factors lead to greatly enhanced thermoelectric performance.

The fabrication of the proposed device is feasible. The recent revolution in graphene research^31^, its synthesis techniques^23^,^24^,^25^ such as electron beam lithography^11^, the use of nanowires as etch mask^32^ and molecular templating have greatly advanced, allowing precise fabrication of GNRs. However, drilling pores in GNRs with atomistic precision is a challenging task. Nevertheless, some recent work has achieved promising advancements towards the realization of smooth-edged GNRs, and such techniques can be implemented for making precise nanopores^33^,^34^,^35^,^36^,^37^. Techniques like transmission electron microscopy^38^,^39^ and Helium Ion beam milling^38^,^40^,^41^ can also be used to drill pores in GNRs with precise control. By exploiting these feasible fabrication techniques it would be possible to realize a GNR-NP based nanoscale thermoelectric device that may form the building block of an extremely localized cooling system, with high device density arrays capable of cooling individual transistor in highly dense integrated circuits. Such a device would also be feasible on flexible substrates. All of these make GNR-NP based thermoelectric devices a promising candidate for future thermoelectronics. In this paper we propose and investigate graphene nanoribbons with nanopores as a new class of planar thermoelectric devices.

## Results

**Figure 1:**
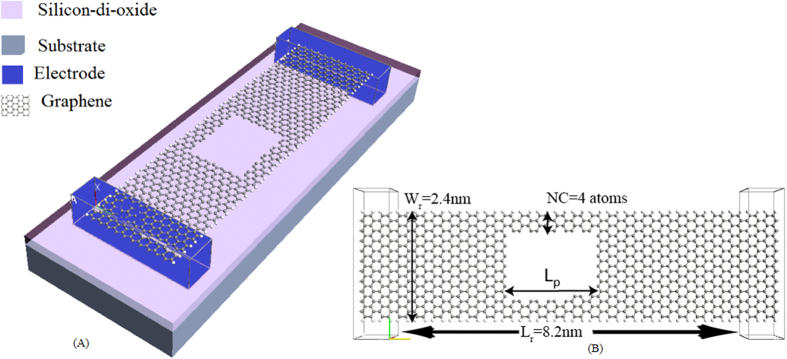
(**A**) Schematic representation of the proposed device. (**B**) Atomistic model of the graphene nanopore thermoelectric device. (L_r_ = Ribbon Length, L_p_ = Pore Length; W_r_ = Ribbon Width; and NC = Side channel width in terms of number of atoms).

An atomistic model of our proposed device structure is shown in Fig. 1. As the figure shows, the device consists of a graphene nanoribbon incorporating a nanopore. For all our simulations the width of our nanoribbon is 2.4 nm (21 atoms wide) and length of our ribbon is 8.2 nm (though the length is varied in later sections). From Fig. 1 we can see that the pore has created two channels on both sides of the nanoribbon. The number of atoms across the width of the side channels (NC) is dependent on the width of the pore. We constructed the pore in such a way that NC remains the same for both side channels. As the electrical property variation can be best explained based on the number of atoms across the width of the side channels (NC), we expressed the width variation in terms of NC.

Based on the configuration described above, there are three important dimensions that need to be investigated and optimized; the overall width of the nanoribbon (W_r_), the width of the side channel (NC) and the length of the pore (L_p_). All of these three parameters are illustrated in Fig. 1(B).

Nanoribbon edges; armchair or zigzag, greatly affect the performance of the device. In this section, we investigate all of these factors through quantum mechanical simulations, and present a strategy for optimizing all of these parameters in order to achieve the highest possible ZT value. The calculation methodology is described in the methods section.

### Zigzag versus Armchair nano-ribbon edges

First we simulated a nanopore device using both zigzag and armchair edged nanoribbons. The device with zigzag nanoribbon edges didn’t exhibit any band gap, which is essential for having a reasonable Seebeck coefficient. The device built with armchair nanoribbon edges showed pore-dimension-dependent transport parameters. In all of our subsequent work we focus on structures constructed with armchair edged nanoribbons. We have kept the width and length of the ribbon constant and varied the pore dimensions.

### Width Dependence

**Figure 2:**
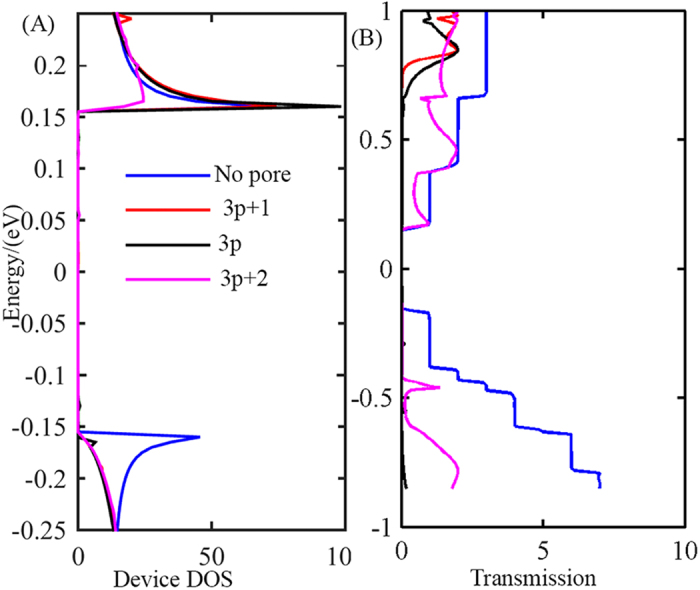
(**A**) Device density of states(DDOS) vs. energy for different pore widths. DDOS without any pore is also included for comparison. (**B**) Transmission vs. energy for the above four cases.

**Figure 3:**
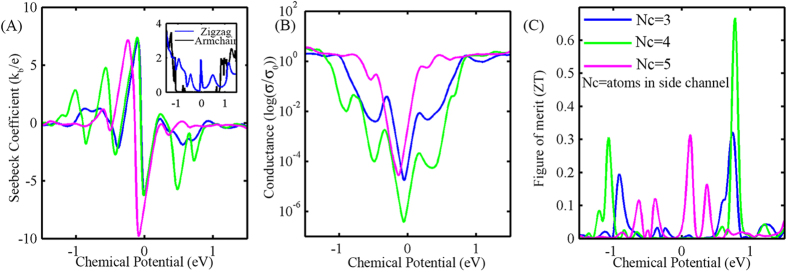
(**A**) Chemical potential (*μ*) dependent Seebeck coefficient (S) for three devices with different side channel widths. Inset: Transmission spectra for zigzag and armchair nano-ribbons. (**B**) Normalized electronic conductance (*σ*/*σ*_0_) for the three devices, where 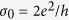 (**C**) thermoelectric figure of merit (ZT) for the three devices. All the calculations are done at T = 300K.

To investigate the effect of pore inclusion and dimensional tuning of its width on the electrical properties of the nanoribbon we plot the device density of states (DDOS) and transmission spectra for a number of devices with different NC values, in Fig. 2(A) respectively. The DDOS plots, in Fig. 2(A) shows that the bandgap remains unchanged (about 0.3 eV) after the inclusion of the pore without any significant change with pore width variation. No significant change was observed at the conductive band edge, with the inclusion of the pore.We found that the conduction band edge is steepest for the devices with NC values of 3p + 1 and 3p. As for the valence band, the valence band edge is steepest for the device with no pore. The trend in the DDOS is reflected in the transmission spectra plots, shown in Fig. 2(B), where the inclusion of the pore significantly changes the shape of the spectra, with the change being most significant for the devices that have an NC value of 3p + 1 and 3p. We then plot the thermoelectric (TE) transport coefficients, as a function of chemical potential, for three devices similar to the one shown in Fig. 1 but with different side channel widths; 3 atoms, 4 atoms and 5 atoms. Within the rigid band model, chemical potential indicates the doping level. In a real device we can achieve different doping levels (or carrier concentrations) by n-type or p-type doping, and in the case of graphene, by applying a varying gate voltage also. To determine the TE transport coefficients, i.e. electrical conductivity and Seebeck coefficient, we first calculate the transmission function T(E). From T(E) we determine the conductivity and Seebeck coefficients based on equations 3, 4, 5(refer to method section). For different pore widths the chemical potential dependence of S, *σ* and ZT is shown in Fig. 3.

For all pore widths, conductance has a sharp rise around the Fermi-level (Fig. 3(B)). Such a large change is reflected in the high Seebeck coefficient (Fig. 3(A)), which, according to Mott’s formula^42^, is the logarithmic derivative of conductance. Figure 3(C) shows the ZT value for different chemical potential values, and suggests that ZT reaches its peak close to the edge of the conduction in transmission spectra (detailed discussion later). For all the devices, we first find the position of the chemical potential that results in a maximum ZT value, and then we extract the value of S and *σ* for that chemical potential.

**Figure 4:**
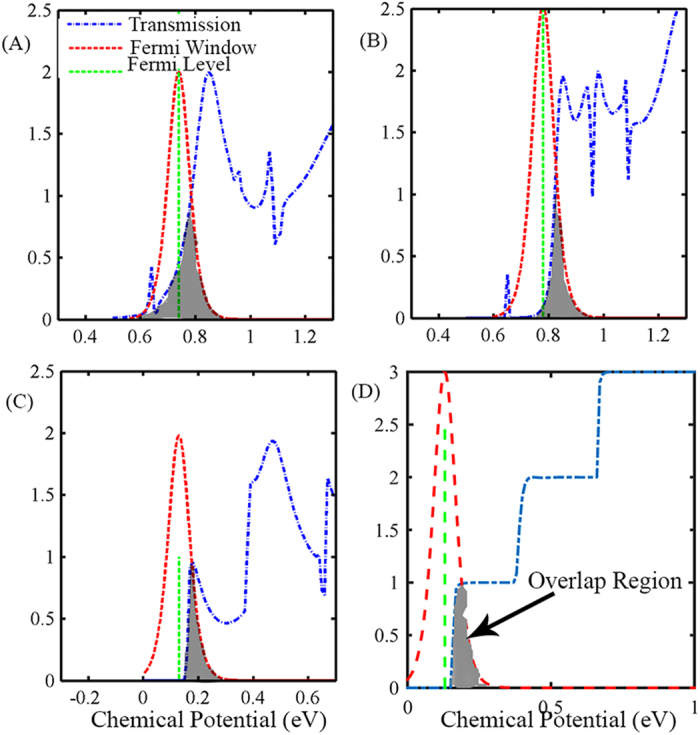
Transmission spectrum at the edge of conduction band along with the position of the Fermi-window for GNR-NP devices with a NC value of (**A**) 3p, (**B**) 3p + 1 and (**C**) 3p + 2 and for a (**D**) GNR with no pore. The overlap is represented by the gray shaded area. Greater overlap between the Fermi-window and the transmission function results in higher conductance, while a greater distance between the Fermi-level and the edge of the conduction band results in a higher Seebeck coefficient.

As equation 3 shows, conductance depends on the overlap of the Fermi-window and the transmission spectra. Accordingly, we can achieve high conductance when we move the chemical potential deep into the conduction band or the valance band of T(E). However, for the Seebeck coefficient, transmission spectrum is weighted by the (E-*μ*) term. This indicates that if chemical potential moves into the conduction band the difference between the energy of the conducting electron and the chemical potential (*μ*) will be low, resulting in small Seebeck coefficient. So there is a trade-off between conductance and Seebeck coefficient which can be manipulated by controlling the shape of the transmission spectra at the onset of conduction^10^. If there is a sharp rise of transmission at some specific energy(Ec) optimum chemical potential will be below Ec. This will result in a high value of Seebeck coefficient because of the (E-*μ*) term, while achieving a reasonable value for conductance due to significant overlap between the tail of the Fermi-window and T(E) around Ec. However, if there is a gradual rise of T(E) at Ec, and we place *μ* below Ec, *σ* will have a very low value due to the small overlap of T(E) with the Fermi-window. To obtain the optimal value, we move *μ* into the conduction band. This results in high *σ* but smaller value of S because of the (E-*μ*) term. These conditions are illustrated for our GNR-NP device when we vary NC, as shown in Fig. 4.

When the value of NC is 3p (where p is an integer), T(E) has a gradual rise at E_c_ (Fig. 4(A)). In this case, the optimal *μ* position is above E_c_, resulting in high *σ* and low S values. While when the value of NC is 3p + 1 and 3p + 2 we get a sharp rise of T(E) at Ec (Fig. 4(B,C)), and hence the optimal *μ* level is just below E_c_, resulting in high S and low *σ* values. In the case of a GNR without any pore, Ec is at low energy and exhibits clear staircase like behaviour. However, the overlap area of transmission spectra and fermi-window at the position where maximum ZT is achieved, is between the 3p + 2 and 3p + 1 cases and smaller than the 3p case. This suggests that it may be possible to not only maintain the conductance of the GNR after pore inclusion, but even further enhance it to higher values if careful dimensional tuning of the nanopore is achieved.

**Figure 5:**
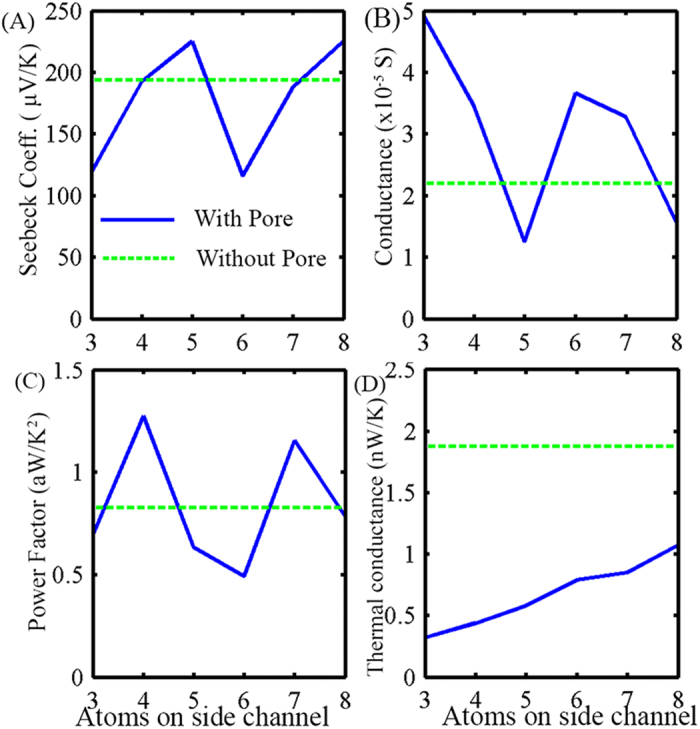
(**A**) Seebeck coefficient, (**B**) conductance, (**C**)power factor (S^2^*σ*), and (**D**) phonon thermal conductance *k*_*ph*_ for GNR-NP devices plotted against the number of atoms in their side channels.

In Fig. 5 we plotted the values of S and *σ* as NC is varied. We can see the periodic behaviour as discussed above, for both S and *σ* with periodicity of 3 atoms. This can be related back to previous work where Son *et al*.^43^ have shown that the bandgap of an AGNR exhibits three different families of behaviour; having largest bandgap when the number of atoms in its width is 3p + 1, smallest when 3p + 2, and in between when 3p (here p is a positive integer). This suggests that when we drill a pore in GNR the side channel behaves like an individual GNR and we can model them accordingly. In Fig. 5, the straight line denotes the value of S and *σ* for a GNR without any pore. This lies between the maximum and minimum achievable value of S and σ when we vary pore dimensions. However, when we plot the power factor (S^2^*σ*), we can achieve better performance than GNRs without a pore, but only when NC is 3p + 1. In addition to the improvement in power factor, GNR-NP also exhibit lower thermal conductivity, as depicted in Fig. 5(D). The straight line indicates the k_ph_ for the GNR without a pore. We can see that the thermal conductivity decreases significantly by introducing nanopores. However, it increases linearly with NC.

**Figure 6:**
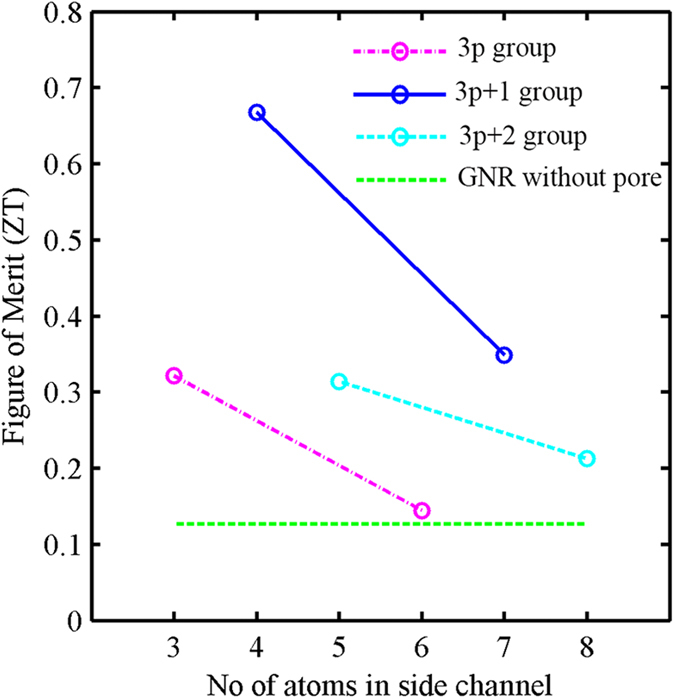
Figure of merit (ZT) for different devices with different numbers of atoms in their side channels. The structures are divided into three groups. Two samples of each group are joined by a straight line. The horizontal line represents the ZT value of GNRs without pores.

Note that with increasing NC, the value of phonon transmission spectra increases. This means that phonons have more paths to flow through, resulting in higher *k*_*ph*_. If we incorporate the improvement in power factor and the reduction in thermal conductivity, we can achieve a ZT that is 6 times higher than conventional GNRs of the same dimension. The improvement of ZT for three groups (3p, 3p + 1 and 3p + 2) is shown in Fig. 6. Each group has two points which are connected by a straight line. We can see a clear dependence of ZT on NC, reaching a peak value when NC is 3p + 1 and a minimum value when NC is 3p (Fig. 6). The downward slope of each group represents the increasing thermal conductivity with NC.

### Length dependence

**Figure 7:**
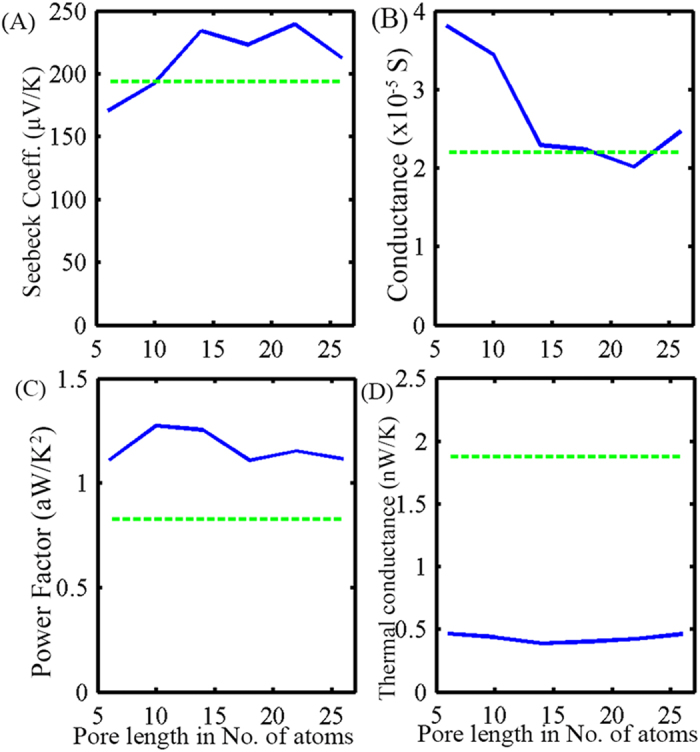
(**A**) Seebeck coefficient (S), (**B**) conductance *σ*, (**C**)power factor(S^2^*σ*), and (**D**) phonon thermal conductance *k*_*ph*_ for different GNR-NP devices with equal pore width (NC = 4) but different pore lengths.

**Figure 8:**
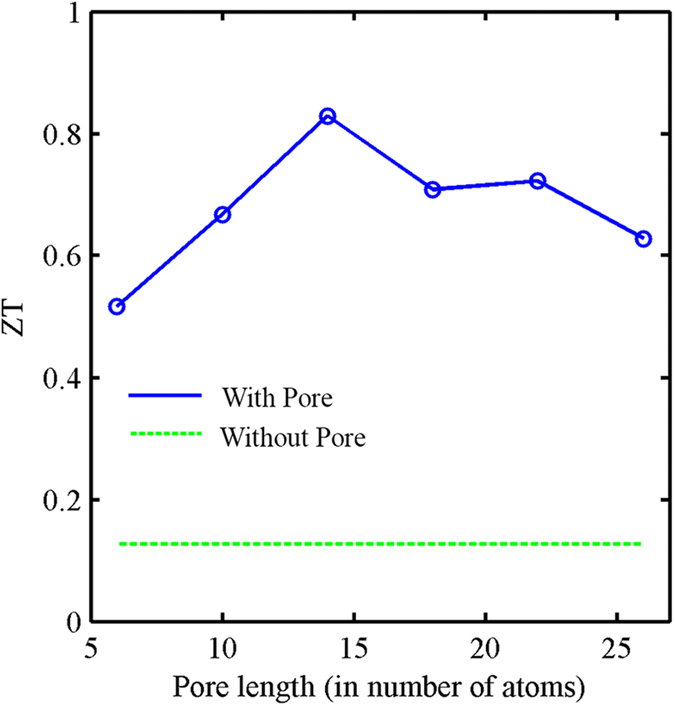
Figure of merit (ZT) for different GNR-NP devices with different pore lengths but with a fixed pore width (NC = 4). The horizontal dashed line represents the ZT value of a GNR without a pore.

In this section we keep the width of the pore fixed at an optimal value (NC = 4) and vary its length. First we conduct a similar type of analysis to the one we conducted when we varied the pore width. For different pore lengths we find the variation of the transport coefficients with the position of the chemical potential, we then extract the value of S and *σ* for the position of *μ* which results in maximum ZT. Figure 7(A) show the variation of S and σ, respectively, with pore length. For small pore lengths the transmission spectrum shows gradual increase at the bottom of the conduction band. This explains the high σ and low S values for the smaller pore lengths (explained before). However, as we increase the pore length, the values of S and *σ* saturate. Here, again, we find a significant improvement in power factor for all pore lengths (Fig. 7(C)). On the other hand, the analysis of *k*_*ph*_ shows no dependence on pore length (Fig. 7(D)). This is consistent with our ballistic model of phonon transport. Combining the improvement in power factor and the reduction in thermal conductivity, we can achieve a ZT that is higher than conventional GNRs of the same dimension for all pore lengths (Fig. 8).

### Temperature dependence

**Figure 9:**
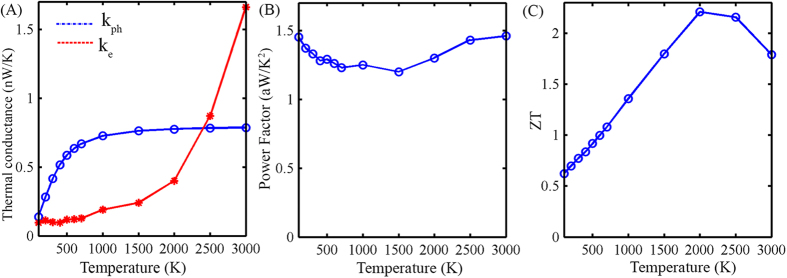
Temperature dependent behavior of (**A**) thermal conductivity due to phonons (k_ph_) and due to electrons (k_e_). (**B**) Power factor and (**C**) Thermoelectric figure of merit (ZT).

As thermoelectric devices usually operate in a wide temperature range, we also analysed the effect of temperature on our device. The phonon thermal conductivity increased with temperature (Fig. 9(A)). This is consistent with other methods used to model phonon thermal conductivity^21^. Moreover, the electrical thermal conductivity also increases with temperature (Fig. 9(A)). On the other hand, the power factor remains almost constant (Fig. 9(B)). However, due to the temperature term in the numerator of ZT, it increases with temperature at low temperature levels and then begins to decrease at very high temperature levels. These calculations are approximate as they don’t take into account the electron-phonon and electron-electron interactions, which can dominate at high temperatures (see methods).

### Device Implementation

**Figure 10:**
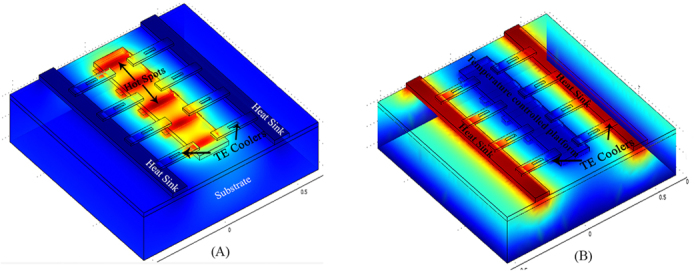
Device concept based on GNR-NP based peltier cooler to (**a**) locally cool hot spots in an IC and (**b**) control the temperature of a platform in the IC. Coolers are connected to a metal line that acts as a heat sink.

Improvement in thermoelectric figure of merit doesn’t necessarily mean a better device with improved thermoelectric heat-to-electricity conversion capability. Current research on thermoelectronics focuses on nanotechnology^9^,^10^,^44^ which requires innovative device strategies to implement them. For example, to implement silicon nanowire as thermoelectric generator Stranz *et al*.^45^,^46^ proposed and implemented a novel thermoelectric device concept. Similarly Davila *et al*.^47^,^48^ proposed a device concept with horizontally aligned nanowires. We propose a similar device concept in Fig. 10, where we use arrays of our GNR based devices to control the temperature of specific regions in integrated circuits (ICs). For example there are certain devices like quantum dots, whose characteristics vary with temperature. Similarly certain nano-biosensors cannot operate outside specific temperature ranges. Using our device concept, which is illustrated in Fig. 10(b), we can precisely control the temperature of the platform where we can fabricate these devices/sensors. Moreover, it is well known that most of the heat generated in integrated circuits (IC) is in the channel region of transistors. By using a GNR based thermoelectric cooler we can extract the heat from the hot spot and release it to nearby heat sink (metal line), as illustrated through Fig. 10(a). However, for this specific application low thermal conductivity is not required because it will impede the heat flow in the required direction. This GNR based thermoelectric cooler and heat sink can be fabricated using the same technology used to fabricate the IC with some additional process steps.

**Figure 11:**
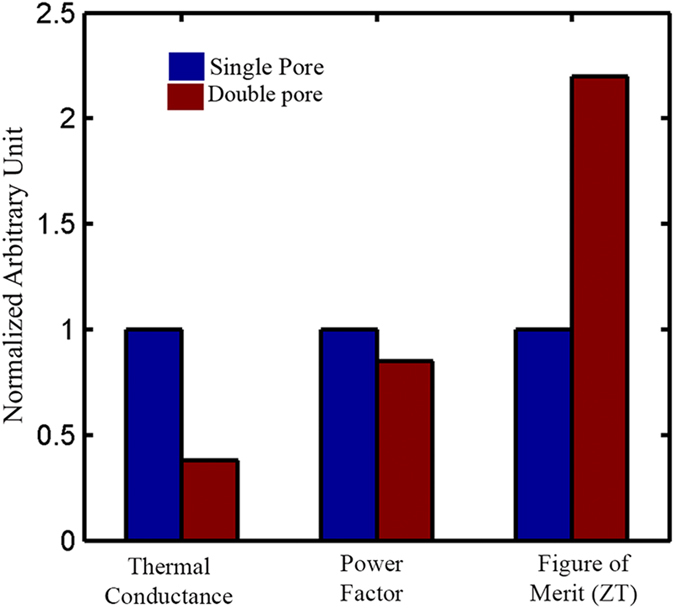
Comparison between single-pore and double-pore GNR based thermoelectric devices. The thermal conductivity reduces significantly with the inclusion of an extra pore. On the other hand, the effect on power factor in not as significant. As a result, ZT almost doubles when compared to the value of a single pore. All the values for the single-pole device are normalised to 1, while the values of the double-pore device are normalized relative to the single-pore device, in order to provide an insightful illustration.

However, as our simulated device was very small in terms of length, we varied the length of our device and found that the length of the ribbon doesn’t play a significant role as long as it remains within the ballistic transport regime. On the other hand, for longer devices multiple pores can be used^22^. To see the effect of increasing the number of pores within the device, we drilled two pores of similar dimensions in our device. Figure 11 shows the comparison between single-pore and double-pore devices. We could see that by increasing the number of pores we can get further reduction in thermal conductivity while maintaining similar electrical transport property (power factor) resulting in an enhancement in the ZT value. From this we can conclude that by using multiple pores (optimized by our strategy) we can get better thermoelectric performance compared to single-pore devices. It is worth mentioning that a single GNR device is insufficient for generating any usable amount of power. However, using top-down approach of nano-fabrication one can fabricate large numbers of these devices connected in series or parallel, which has the potential to produce useful amounts of power.

## Discussion

In summary, we have investigated the relationship between different pore dimensions and the thermoelectric performance of GNR-NP devices. Our analysis shows that the electrical performance of the nano-device depends greatly on the width of the side channels. We are able to modulate the transmission spectra by varying the number of atoms across the width of the side channel. A sharp increase of T(E) at the onset of the conduction band resulted in the best device performance. On the other hand, the effect of pore length was less pronounced on both electrical and thermal performance. By using the optimal values for the different pore dimensions we could reach a ZT value that is 6 times higher than a GNR without a pore. Here, we did not use long GNRs in order to lower the thermal conductivity, instead, we focused on the transmission spectra and how it can be modified to achieve better thermoelectric performance. Our investigation here shows that graphene can be used to realize thermoelectric devices with nanoscale dimensions, which could enable the realization of large density arrays of such devices or even the integration of individual thermoelectric devices with individual electronic devices such as a transistors. It is worth noting that we have made some simplifications in our used model, including neglecting electron-phonon interactions, which may result in quantitative discrepancy from experimental values, however such simplifications are justifiable (discussed in the methods section in more details) and should not result in significant qualitative discrepancy. Nevertheless, our analysis shows a route towards achieving better thermoelectric performance in GNR devices by incorporating Nanopores and tuning their dimensions. The experimental realization of the devices will require precise control of the edge structure of GNRs, and with the introduction of advanced fabrication techniques this precision will be available in the near future^33^,^35^,^40^.

## Methods

The measure of performance of a thermoelectric material is given by the dimensionless figure of merit:


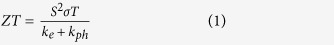


Where S is Seebeck Coefficient, *σ* is electrical conductance and T is temperature. Thermal conductance consists of two parts, thermal electron conductance *k*_*e*_, and thermal phonon conductance *k*_*ph*_.

Assuming a linear response regime, the thermoelectric properties of a nanosystem can be determined from the definition of the electrical and thermal currents:


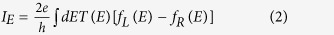



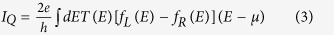


Here, *e* is the carrier charge, *T*(*E*) is the transmission coefficient of the device, *f*_*L*_ and *f*_*R*_ are the distribution function of left and right reservoirs with chemical potentials *μ*_*L*_ and *μ*_*R*_. As we are interested in the linear response regime, assuming Δ*μ* and ∆T as infinitesimally small quantities, chemical potential *μ* is written as the average of left and right electrode chemical potentials. Now, by using Taylor expansion of equations 2 and 3 we can obtain the thermoelectric transport properties as below:


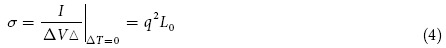



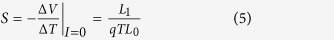



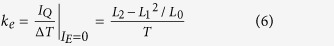


Where *L*_*n*_ (n = 0,1,2) is:


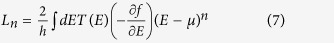


The formalism used here is adopted from the work of Esfarjani *et al*.^49^. Here, 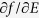 is the derivative of the Fermi function. *T*(*E*), the transmission coefficient, is calculated from the Hamiltonian H and overlap matrices S_ov_ using NEGF formalism. The device’s H and S_ov_ matrices were calculated using the Extended Huckel technique^50^. The device structure is partitioned into three regions: semi-infinite left electrode, central scattering region and semi-infinite right electrode. The mesh points in real space calculation were defined as 1 × 1 × 100 k points.

To calculate the phonon thermal conductance we used the Landauer approach^51^:


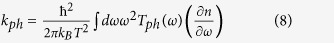


The phonon transmission T_ph_(ω) is calculated in a similar technique to the one used for calculating the electrical conductance, by substituting H by a force constant matrix K, and substituting S_ov_ by a diagonal matrix of atomic mass M. The term 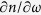 is the derivative of the Bose-Einstein distribution for phonons. To calculate the force constant matrix for phonons, empirical Tersoff potential was used. Both simulations were implemented in the Atomistix Toolkit (ATK) software package^52^. Prior to calculation, each device was optimized and its co-ordinates were relaxed until Tersoff potential forces on individual atoms were smaller than 0.01 eVȦ^−2^.

In this work we have performed independent electron and phonon calculations, and hence electron-phonon interactions are neglected. This simplification can be justified based on the work by Chen *et al*.^53^ where they have experimentally showed that the mean free path for electron-acoustic phonon scattering in graphene is greater that 2 *μ*m, which is much larger than our device dimensions.

## Additional Information

**How to cite this article**: Sharafat Hossain, M. *et al*. High Performance Graphene Nano-ribbon Thermoelectric Devices by Incorporation and Dimensional Tuning of Nanopores. *Sci. Rep*. **5**, 11297; doi: 10.1038/srep11297 (2015).
